# Neuropsychological functioning of individuals at clinical evaluation of adult ADHD

**DOI:** 10.1007/s00702-020-02281-0

**Published:** 2020-12-23

**Authors:** Nana Guo, Anselm B. M. Fuermaier, Janneke Koerts, Bernhard W. Mueller, Katerina Diers, Aaron Mroß, Christian Mette, Lara Tucha, Oliver Tucha

**Affiliations:** 1 Department of Clinical and Developmental Neuropsychology, Faculty of Behavioral and Social Sciences, University of Groningen, Grote Kruisstraat 2/1, 9712 TS Groningen, The Netherlands; 2 Department of Psychiatry and Psychotherapy, Faculty of Medicine, LVR Hospital Essen, University of Duisburg-Essen, Essen, Germany; 3 Department of Psychology, University of Wuppertal, Wuppertal, Germany; 4 Protestant University of Applied Sciences, Bochum, Germany; 5 Department of Psychiatry and Psychotherapy, University Medical Center Rostock, Rostock, Germany

**Keywords:** Adult ADHD, Neuropsychology, Cognition, Assessment, Diagnosis

## Abstract

**Objectives:**

Numerous studies showed that adults with attention deficit hyperactivity disorder (ADHD) suffer from impairments in a range of cognitive functions when compared to healthy controls. However, only little is known about the neuropsychological functions when compared to various clinical control groups and whether a distinct neuropsychological profile can be identified for adult ADHD.

**Method:**

This retrospective study examined data of 199 outpatients referred for clinical evaluation of adult ADHD, allocated either to an ADHD group (*n* = 78) or to one of two clinical comparison groups, depending on whether they show indications (*n* = 71) or no indications (*n* = 50) for the presence of psychiatric disorders other than ADHD. All individuals performed a comprehensive neuropsychological test battery.

**Results:**

Data analysis revealed impairments in a range of cognitive functions in a substantial number of patients of all three groups. However, profiles of neuropsychological impairments were similar between groups. Furthermore, significant small- to medium-sized correlations between basic and higher-order cognitive functions were revealed in the ADHD group and the clinical comparison group with indications for psychiatric disorders other than ADHD.

**Conclusion:**

Neuropsychological impairments are prominent in psychiatric outpatients seeking a clinical evaluation of adult ADHD but are not specific for ADHD. It is concluded that neuropsychological test performance may have limited incremental value to support the psychiatric differential diagnosis. Furthermore, a clinical trajectory may need to take into account that deficits in a range of higher-order cognitive functions can be substantially explained by deficits in basic cognitive functions.

## Introduction

Attention deficit hyperactivity disorder (ADHD) is one of the most prevalent neurodevelopmental childhood disorders that persists into adulthood in a large proportion of cases (Biederman, Petty, Clarke, Lomedico, and Faraone 2011; Polanczyk, De Lima, Horta, Biederman, and Rohde 2007; Stubbe 2000; Weiss and Hechtman 1993). ADHD is characterized by symptoms of inattention, hyperactivity, and impulsivity (American Psychiatric Association, 2013; Barkley 2006). A range of functional impairments are associated with ADHD in adulthood when compared to healthy controls, mainly including lower educational attainment and employment rate (Biederman 2005; Faraone et al. 2000; Gjervan et al. 2012; Sobanski et al. 2007; Holst, and Thorell 2019), poorer financial situation (Bangma et al. 2019; Biederman et al. 1993), lower self-esteem (Canu and Carlson, 2007), more alcohol and drug abuse (Torgersen, Gjervan, and Rasmussen, 2006; Cumyn, French, and Hechtman, 2009), and a lower quality of life (Agarwal, Goldenberg, Perry, and Ishak 2012; Stern et al. 2017).

Because ADHD is by definition a disorder with predominant cognitive dysfunctions that interfere with many tasks of daily living, a large body of neuropsychological research has been performed to elucidate the level of neuropsychological functioning of individuals with ADHD. Converging evidence from numerous studies revealed impairments of adults with ADHD in multiple domains of cognition, including different aspects of attention, processing speed, memory and executive functions (Barkley and Murphy 2010; Boonstra et al. 2005; Brown, 2002; Fuermaier et al. 2015; Jacobson et al. 2011; Tucha et al. 2009). Research further revealed that neuropsychological functions appear to improve, but do not normalize under pharmacological treatment with stimulants, as deficits are still present under stable medication especially in the domains of memory and attention (Fuermaier et al. 2017; Muller et al. 2007). Furthermore, studies showed that the impairments in the various domains of cognition may not be independent entities, but that impairments in basic cognition, such as processing speed and attention focus, may explain a considerable proportion of the impairments in the more complex cognitive functions, such as divided attention, memory, or executive functions (Holst and Thorell 2017; Boonstra et al. 2010). Due to the cognitive impairments of adults with ADHD, the assessment of neuropsychological functions using cognitive performance tests has been suggested to be of added value to the clinical evaluation of adults with ADHD. In this respect, neuropsychological assessments are performed to characterize individual cognitive strengths and weaknesses, which may help to understand why an individual patient is experiencing problems in daily life (Barkley, and Fischer 2011; Mapou 2019; Stern et al. 2017; Yáñez-Téllez et al. 2012).

However, defining the role of a neuropsychological assessment in the clinical evaluation of adult ADHD is complicated because of the large heterogeneity of findings in previous research. For example, although adult ADHD was found in numerous studies to be associated with multiple cognitive impairments on a group level, not all adults with ADHD share the same type and degree of cognitive impairment, with some patients even showing not a single cognitive impairment in a cognitive test battery (Mostert et al. 2015; Nigg, Willcutt, Doyle, and Sonuga-Barke 2005; Wåhlstedt, Thorell, and Bohlin 2009). The heterogeneity of findings does not allow clear conclusions about what functions are more helpful in discriminating patients affected with ADHD from individuals not being affected with ADHD within a clinical evaluation (Dias et al. 2013). This heterogeneity is also reflected in a recent consensus report including international renowned experts in the field, which suggests as many as 16 cognitive functions to be relevant in a clinical neuropsychological assessment of adults with ADHD (Fuermaier et al. 2019).

Moreover, the majority of previous studies revealed cognitive differences between adults diagnosed with ADHD and healthy control group as recruited from the local community (Boonstra et al. 2005; Alderson et al. 2013). This comparison may not be representative for the use of neuropsychological assessment in the evaluation of ADHD in clinical practice, where individuals with ADHD are sought to be differentiated from clinical controls, which include individuals having other psychiatric conditions or individuals who do not reach diagnostic criteria for any psychiatric disorder but nevertheless had reasons for referral. In this respect, Holst and Thorell (2017), Pettersson, Soderstrom, and Nillson (2018) as well as Braek, Dijkstra, and Jolles (2011) found that patients with ADHD performed significantly poorer in a range of neuropsychological tasks compared to a clinical control group in an ADHD outpatient assessment, including measures of reaction time variability, attention, vigilance, inhibition, verbal (working) memory, verbal learning, set shifting, planning, fluency, and delay aversion. However, effect sizes of group differences were mostly small to moderate, and neuropsychological tests were found to have a relatively poor ability to discriminate between adults with ADHD and clinical controls. In another study, Wiig and Nielsen (2012) revealed participants with ADHD to be significantly slower in a task for processing speed than both a healthy and a clinical control group, whereas no significant differences were observed between these two control groups. In contrast to the findings differentiating adults with ADHD from clinical controls, Walker and colleagues (2000) could only demonstrate cognitive impairments of adults with ADHD when compared to a healthy control group, but not when compared to a clinical control group. Similarly, Marchetta, Hurks, Krabbendam, and Jolles (2008) reported a range of cognitive impairments of adults with ADHD when compared to a healthy control group, but significant difference to a clinical control group was found only in a task for mental flexibility. Given these findings, it can be concluded that studies comparing cognitive functions between patients with ADHD and relevant clinical control groups in the same clinical setting are still scarce and that findings across studies remain inconsistent (In de Braek et al. 2011; Holst and Thorell 2017; Marchetta et al. 2008; Pettersson et al. 2018; Walker et al. 2000; Wiig and Nielsen 2012).

Due to the heterogeneity of the applied research (including differences in patient samples, control groups and cognitive measures applied), conclusions about what cognitive impairments are most characteristic for ADHD are difficult to draw. Thus, in order to further elucidate the role of a neuropsychological assessment in the clinical evaluation of adult ADHD, the present study employs a large sample of clinically referred individuals to an ADHD outpatient clinic (*n* = 248), who all performed a comprehensive test battery consisting of a broad range of measures, which was specifically composed for the neuropsychological assessment of adult ADHD. In this study, we aim to reveal differences in cognitive functions between individuals who receive a diagnosis of ADHD and individuals who have been referred for clinical assessment because of an assumed ADHD, but who actually did not fulfill the diagnostic criteria of an ADHD. We expect that adults diagnosed with ADHD perform significantly poorer in several aspects of attention and executive function than individuals not reaching diagnostic criteria for ADHD. However, we expect that effect sizes of impairments between groups differ across functions and will not exceed small to medium size (Boonstra et al. 2005; Hervey et al. 2004; Marchetta et al. 2008; Mostert et al. 2015; Pettersson et al. 2018). Moreover, as motivated by previous findings (Butzbach et al. 2019; Holst and Thorell 2017; Boonstra et al. 2010), we aim to quantify the effect of basic cognitive functions (i.e., processing speed and distractibility) on more complex cognitive functions (i.e., different aspects of complex attention and executive control) in adults with ADHD and seek to determine whether this hierarchical relationship is shaped differently in groups not having ADHD, such as being diagnosed with other psychiatric disorders or did not fulfill the diagnostic criteria of any psychiatric disorders.

## Method

### Participants

Two hundred and forty-eight participants were considered for inclusion in the present study. All participants were suspected to have ADHD (e.g., by general practitioners, neurologists, or psychiatrists) and were therefore referred for a diagnostic assessment to the ADHD outpatient clinic of the Department of Psychiatry and Psychotherapy, University of Duisburg-Essen, Germany. All individuals underwent a comprehensive diagnostic assessment by trained psychologists or psychiatrists. The diagnosis of ADHD was established based on the criteria as outlined in the Diagnostic and Statistical Manual of Mental Disorders, 5th Edition (DSM–5; American Psychiatric Association 2013). The assessment procedure included a semi-structured interview to evaluate ADHD psychopathology (i.e., the Wender–Reimherr Interview, Retz-Junginger, Giesen, Philipp-Wiegmann, Rösler and Retz 2017; and the Essen-Interview-for-school-days-related-biography, Grabemann et al. 2017). Furthermore, two self-report scales were completed by all participants to quantify the retrospective and current ADHD symptom severity (Rösler et al. 2008). The German version of the Wender Utah Rating Scale (WURS-K) was used to evaluate the retrospective symptoms in childhood (Retz-Junginger et al. 2003; Ward et al. 1993), while the German version of the ADHD Self-Report Scale (ADHS-SB) was administered to assess current ADHD symptoms (Adler et al. 2006; 2008; Kessler et al. 2005; Rösler et al. 2008). The diagnostic evaluation also included objective measures such as evidence derived from school reports and reports of failure in academic and/or occupational achievement, and comprised multiple informants for all individuals (e.g., employer evaluation, partner or parent-reports). The neuropsychological assessment using cognitive tests was part of the routine examination of all individuals in the ADHD outpatient clinic, however, cognitive test results were not part of the standard diagnostic decision process and decision-making. All individuals agreed to their data being used for scientific purposes and gave written informed consent.

Forty-nine of the 248 participants were excluded from the present study, i.e., 47 participants were excluded because the diagnostic process was not completed or did not allow a formal diagnostic decision, and two participants were not considered because the neuropsychological assessment was not or only partly administered, leaving a sample of 199 participants for inclusion in the final data set that entered data analysis. All participants in this sample were allocated to one of three diagnostic groups, i.e., the ADHD group (diagnosis of ADHD was established, *n* = 78), the Clinical Comparison Group (CCG; participants did not meet diagnostic criteria for ADHD but showed evidence for one or more other psychiatric disorders; *n* = 71) and the Cinical Comparison Group-Not Diagnosed (CCG-ND; participants did not meet diagnostic criteria for ADHD and were also not diagnosed with any other psychiatric disorder; *n* = 50). Participants of the CCG showed evidence for one or more psychiatric disorders other than ADHD, including mood disorders (*n* = 50), addiction disorders (*n* = 22), anxiety disorders (*n* = 5), personality disorders (*n* = 3), eating disorders (*n* = 3), adjustment disorders (*n* = 2), schizoaffective disorders (*n* = 2), obsessive–compulsive disorders (*n* = 1), conduct disorders (*n* = 1), and intellectual developmental disorders (*n* = 1). With regard to symptom presentations of ADHD, 66 patients with ADHD were diagnosed with the combined presentation and nine patients with the predominantly inattentive presentation, whereas the symptom presentation of three other patients with ADHD was not reported. Moreover, 31 of the 78 patients with ADHD showed evidence for one or more comorbid psychiatric disorders, including mood disorders (*n* = 19), addiction disorders (*n* = 5), adjustment disorders (*n* = 5), anxiety disorders (*n* = 3), obsessive–compulsive disorders (*n* = 2), personality disorders (*n* = 1), oppositional defiant disorders (*n* = 1), intellectual developmental disorders (*n* = 1), and autistic disorders (*n* = 1). Table 1 presents characteristics of the three groups (ADHD, CCG, CCG-ND) and revealed significant group differences in age, *F*(2) = 7.026, *p* = 0.001, sex, *χ*^2^(2) = 6.553, *p* = 0.038, education level, *χ*^2^(8) = 16.718, *p* = 0.033, childhood ADHD symptoms, *F*(2) = 24.486, *p* < 0.001, and current ADHD symptoms, *F*(2) = 12.060, *p* < 0.001. Compared to the CCG-ND, patients with ADHD had a significantly lower female-to-male ratio, and scored significantly higher on childhood and current ADHD symptoms. Compared to the CCG, patients with ADHD were on average significantly younger, more individuals attained a relatively low level of education, and obtained significantly higher scores in both scales for ADHD symptom severity. The CCG only differed significantly from the CCG-ND with regard to a higher score for childhood ADHD symptoms.

**Table 1:** Characteristics (M ± SD) of the ADHD group (ADHD), clinical comparison group (CCG), and clinical comparison group-not diagnosed (CCG-ND)

	ADHD (*n* = 78)	CCG (*n* = 71)	CCG-ND (*n* = 50)	*F*/*χ*^2^	*p* value
Age (in years)	31.9 ± 10.3^b^	38.8 ± 11.2	35.4 ± 12.1	7.026	0.001
Sex (female/male)	27/51^a^	28/43	28/21^6^	6.553	0.038
Education (1/2/3/4/5)^1^	4/23/15/22/13^6 b^	0/11/29/18/12^6^	0/14/12/16/8	16.718	0.033
Childhood ADHD symptoms^2^	44.6 ± 12.4^a b^	34.5 ± 11.4^a^	27.9 ± 12.5	26.486	< 0.001
Current ADHD symptoms^3^	35.5 ± 9.8^a b^	29.6 ± 8.7	26.4 ± 11.7	12.060	< 0.001
Symptom presentation of ADHD^4^	66/9/0/3				
Psychiatric disorders other than ADHD^5^	19/5/3/1/0/5/0/2/1/1/1	50/22/5/3/3/2/2/1/1/1/0			

*ADHD* attention deficit Hyperactivity disorder; *CCG* clinical comparison group; *CCG-ND* clinical comparison group-not diagnosed

^1^Education (1/2/3/4/5) = no school-leaving qualification/compulsory schooling or intermediate secondary school/college or vocational training/Higher secondary school with university entrance qualification/university

^2^Childhood ADHD symptoms as measured with the German version of the Wender Utah rating scale-short version

^3^Current ADHD symptoms as measured with the German version of the ADHD self-report scale

^4^Symptom presentation of ADHD = combined/inattentive/hyperactive-impulsive/not reported

^5^Psychiatric disorders other than ADHD = mood disorders/addiction disorders/anxiety disorders/personality disorders/eating disorders/adjustment disorders/schizoaffective disorders/obsessive–compulsive disorders/conduct disorders/intellectual developmental disorders/autistic disorders

^6^Sex/education was not reported in one case

^a^*p* < .05 when compared with CCG-ND

^b^*p* < .05 when compared with CCG

## Measures

### Self-report scales for ADHD symptom

The German version of the Wender Utah Rating Scale (WURS-K) was administered to assess childhood ADHD symptoms retrospectively (Retz-Junginger et al. 2003; Ward et al. 1993). The WURS-K includes 25 items, each answered on a 5-point Likert scale. The German version of the ADHD self-report scale (ADHS-SB, Adler et al. 2006; Kessler et al. 2005; Rösler et al. 2008) was used to quantify the severity of current ADHD symptoms. The ADHS-SB consists of 18 items, each answered on a 4-point Likert scale. A sum score was calculated for each scale.

### Neuropsychological tests for cognitive functions

The test battery Cognitive Functions ADHD (CFADHD; Tucha et al. 2013) of the Vienna Test System (VTS, Schuhfried 2013) was administered to all participants. The CFADHD is a computerized test battery assessing cognitive functions in which adults with ADHD have been shown to commonly present difficulties.

### Selective attention

The WAFS (Perceptual and Attention Functions-Selective Attention, Sturm 2011) is administered to assess selective attention. In this test, a total of 144 stimuli (circle, triangle or square) were consecutively presented in the center of the computer screen, which will get lighter or darker or remain the same. The changes in circles and squares were defined as the target (30 targets). Participants were asked to press a response button as quickly as possible whenever a target (i.e., a circle gets lighter, a circle gets darker, a square gets lighter, or a square gets darker) was presented, and withhold a response if the target was not shown. The mean reaction time (RT in milliseconds) and dispersion of reaction time (SDRT) were registered. Moreover, the number of omission errors was recorded.

### Vigilance

Vigilance is measured with the WAFV (Perceptual and Attention Functions-Vigilance, Sturm 2012) of the VTS. In this test, a total of 900 squares were consecutively presented to the participants. A target is defined if the presented square becomes darker in shading (50 targets in total). Participants have to press a specific response button as quickly as possible when a target event occurs. The mean reaction time (RT in milliseconds) is registered. Moreover, the number of omission errors is recorded.

### Working memory

A variant of the N-back task as originally introduced by Kirchner (1958) was administered as a test for working memory, i.e., the 2-back version of the N-back verbal task (NBV, Schellig and Schuri, 2012). A total of 100 consonants are consecutively presented to participants. Participants are asked to respond to each consonant that is identical to the last-but-one (e.g., F–K–G–H–B–L–B–S). The number of correct responses is recorded.

### Figural fluency

Figural fluency is measured with the 5-Point Test—Langensteinbach Version (Rodewald et al. 2014), which is based on the task paradigm of the Design Fluency Test (Jones-Gotman and Milner 1977). An input field in the lower half of a divided screen is presented to participants, in which five symmetrically arranged dots are given. Participants are asked to create as many different patterns as possible in 2 min by connecting at least two dots. Dots can be connected by clicking on the space between two dots. All patterns that have been created are presented in the upper half of the divided screen. The total number of unique patterns created in 2 min is recorded.

### Interference

Interference is assessed with the Stroop Interference Test (Schuhfried 2016). This test is a variant of the color–word interference, which was introduced by Stroop (1935) as a measure of interference function. This test contains four conditions. The first condition is a color–word condition, in which color–words (BLUE, GREEN, YELLOW, RED) printed in gray are shown on the computer screen and participants are asked to press the button of the same color as the meaning of the color word. The second condition is a color–banner condition, in which colored banners (banners printed in blue, green, yellow and red) are presented. Participants are asked to press the button of the same color as the color of banners. The third condition is a reading-interference condition, in which color–words (BLUE, GREEN, YELLOW, RED) are printed in mismatching ink (e.g., BLUE printed in green ink). Participants are required to press the button of the same color as the meaning of the color word, ignoring the color the word was printed. The fourth condition is a naming-interference condition, which is analog to the reading-interference condition in which color–words are presented in mismatching ink (e.g., RED printed in blue ink). Participants are asked to press the button of the same color as the ink of the word. Participants are asked to respond as thoroughly as possible, but at the same time as quickly as possible throughout the test. The main variables of interest are reading interference and naming interference. *Reading interference* is calculated by subtracting the time needed for completing the color–word condition from the time needed for the reading-interference condition. *Naming interference* is calculated by subtracting the time needed for completing the color–banner condition from the time needed for the naming-interference condition.

### Processing speed and flexibility

The Trail Making Test—Langensteinbach Version (TMT-L, Rodewald et al. 2012) is administered as a test for processing speed and flexibility. The TMT-L is closely oriented on the Army Individual Test Battery (1944) and the original form of the Trail Making Test by Reitan (1958). The TMT-L consists of two parts. In part A, the numbers 1–25 are simultaneously presented on the screen and participants are asked to join the numbers in ascending order as quickly as possible by clicking on them. In part B, the numbers of 1–13 and the letters of A–L are presented, and participants are requested to connect numbers and letters alternately in ascending order as quickly as possible (i.e., 1-A-2-B-3-C…). The times needed for part A and part B are registered. Part A is used as a measure of processing speed. Flexibility is assessed by the quotient of the times needed for part B by part A.

### Planning ability

Planning ability is assessed with the Tower of London—Freiburg Version (TOL-F, Christoph et al. 2011) of the VTS. The TOL-F dates back to the design originally proposed by Shallice to measure planning ability (Shallice 1982). The task requires participants to move balls of different colors (red, yellow, blue) that can be placed on three rods from given positions to certain target positions. Start state and goal state are presented on the lower and upper part of the computer screen, respectively. The left rod can hold three balls, the middle one can hold two, and the right one can hold only one. Participants are asked to convert a given start state into a goal state by using the minimum number of moves possible. The minimum number of moves to convert a given start state into a goal state is shown on the left of the screen. The item that is being worked on is automatically terminated after 60 s. If it has not been solved within this time, the next item will be presented. A total of 28 items are included in the test and presented in the order of an increasing minimum number of moves. The number of items solved in the minimum number of moves is registered.

### Inhibition

Inhibition is assessed with a Go/No-Go test paradigm (Kaiser et al. 2016), as originally designed for the measurement of inhibitory control (Drewe 1975). In this test, a series of triangles and circles are consecutively presented on the screen. Participants are asked to press a response button when a triangle is presented and to show no response to a circle stimulus. A total of 250 stimuli (202 triangles, 48 circles) are presented in the test, each for 200 ms. The interstimulus interval is one second. The number of commission errors is recorded.

### Task switching

Task switching is assessed with the SWITCH (Gmehlin et al. 2017) of the VTS. In this test, a series of visual stimuli with different forms (circle or triangle) and different brightness (light or dark) are consecutively presented. Participants are asked to respond to stimuli based on two rules that are applied alternately. One rule asks participants to react to form (circle or triangle) but ignore brightness. The other rule requires participants to react to brightness (light or dark) but ignore form. After every two stimuli, participants must change whichever rule is being applied and apply the other rule. The tasks requiring the same rules as used in the last are defined as repeated tasks and tasks requiring different rules as used in the last are defined as switch tasks. The main variable of interest is *task switch accuracy*. *Task switching accuracy* is calculated by subtracting the number of correct responses in switch tasks from the number of correct responses in repeated tasks.

### Subjective experiences of cognitive functioning

The *Questionnaire on Mental Ability* (FLEI; Beblo et al. 2012) as part of the CFADHD on the VTS was administered to assess self-reported cognitive deficits. Items of this questionnaire ask participants to indicate to which extent everyday manifestations of problems in attention, executive functioning, and memory, apply to them. The FLEI includes 35 items scored on a 5-point Likert scale ranging from 0 (never) to 4 (very often). A sum score is computed to indicate the self-reported cognitive deficits.

## Procedure

The diagnostic and neuropsychological assessments were both part of the standard clinical routine of all participants referred to the ADHD outpatient clinic of the University of Duisburg-Essen, Germany. All participants agreed and signed a written informed consent for their data being used for scientific purposes. Ethical approval for this procedure was provided by the local ethical review board (20-9380-BO). Participation was voluntary, unpaid, and it was stressed that the agreement to take part in research did not affect their clinical assessment or treatment. All participants were asked to complete a set of questionnaires at home prior to the diagnostic interview. The clinical evaluation started with the diagnostic interview, and continued with the neuropsychological assessment (cognitive testing) at the same or another day of convenience for the examinee. The neuropsychological assessment using cognitive tests took about two hours to administer, and was led by a trained psychologist or neuropsychological test assistant under close supervision. Participants were not informed about their diagnostic status at the time of the neuropsychological assessment.

### Statistical analysis

Missing values occurred in 5.3% of the data due to administrative errors and were not replaced. Test data of all three groups are presented in descriptive statistics. Furthermore, neuropsychological test data are interpreted based on norm scores as provided by the test publisher, i.e., to derive the number of individuals having impairment in each of the functions assessed. An impairment is defined as an individual test performance that is equal or below the 16th percentile (i.e., one SD below the mean) of the representative test norms as provided by the test publisher (Schuhfried 2013).

Furthermore, neuropsychological functions are compared between groups using statistical significance tests and effect sizes. Because assumptions for parametric analyses (e.g., normality, homogeneity of variances) were not met in several variables, nonparametric statistical analyses were performed. Per test score, the ADHD group was compared with the CCG and CCG-ND, respectively, using Mann–Whitney *U* tests. The significance level was adjusted to *p* < 0.01 in order to control for alpha error growth in multiple testing. The effect size Cohen’s *r* was calculated to indicate the magnitude of pairwise group differences. Cohen’s *r* was chosen as it does not rely on the normality assumption. Based on Cohen’s criteria for *r*, 0.1 indicates a small effect, 0.3 indicates a medium effect, and 0.5 indicates a large effect (Cohen 1988).

In order to investigate the effect of basic on complex cognitive functions, functional domain scores were created representing different aspects of basic (i.e., processing speed and distractibility) and complex (i.e., different aspects of complex attention and executive control) cognitive functions (see Table 4). Basic cognitive functions were measured with the variables of the selective attention task (logarithmic mean of RT, logarithmic standard deviation of RT, omissions), vigilance task (logarithmic mean of RT and omissions) and TMT part A. With regard to complex cognitive functions, it is differentiated between working memory (NBV correct responses), inhibition/interference control (Go/No-Go omissions, Stroop Interference Test naming interference and reading interference), cognitive flexibility (TMT part B/TMT part A, SWITCH task switch accuracy), fluency (number of unique patterns created), and planning (TOL-F number of items solved). All test variables per defined functional domain are z-standardized based on scores of the CCG-ND and averaged in order to obtain one measure per functional domain. In addition, the association between basic cognitive functions and each aspect of complex cognitive functions is examined by Spearman rank correlation coefficients, separately for the ADHD group, CCG, and CCG-ND. The size of the association is interpreted as small (*r* = 0.1), medium (*r* = 0.3), and large (*r* = 0.5).

## Results

**Fig. 1:**
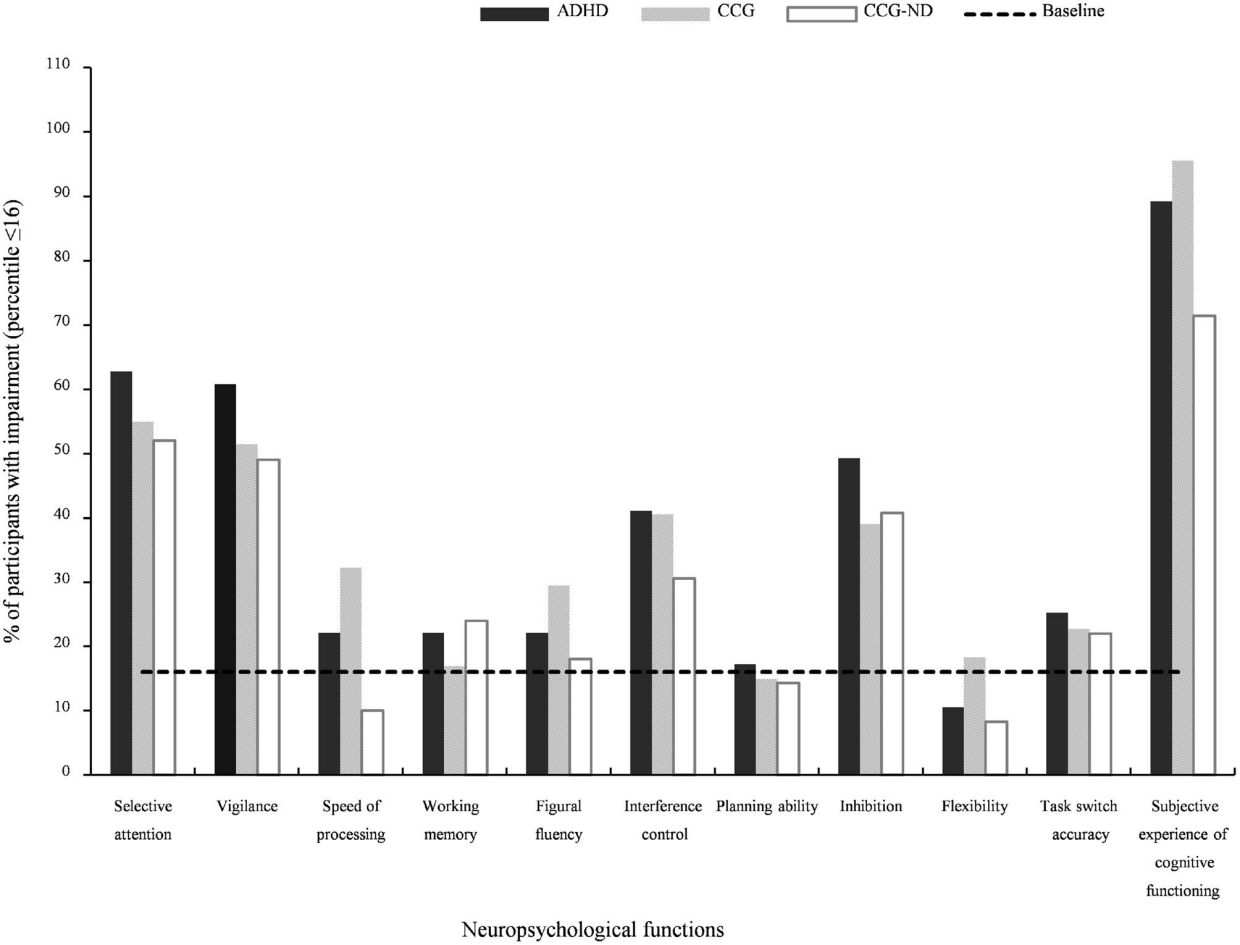
Percentage of individuals indicating impairment (percentile rank ≤ 16) in neuropsychological test performance and self-report. Impairment per function is defined if test performance is impaired in at least one test variable of this function; Dotted line indicates 16% of participants having impairment (i.e., baseline if impairment is defined as percentile rank ≤ 16); *ADHD* attention deficit hyperactivity disorder; *CCG* clinical comparison group; *CCG-ND* clinical comparison group-not diagnosed

**Fig. 2:**
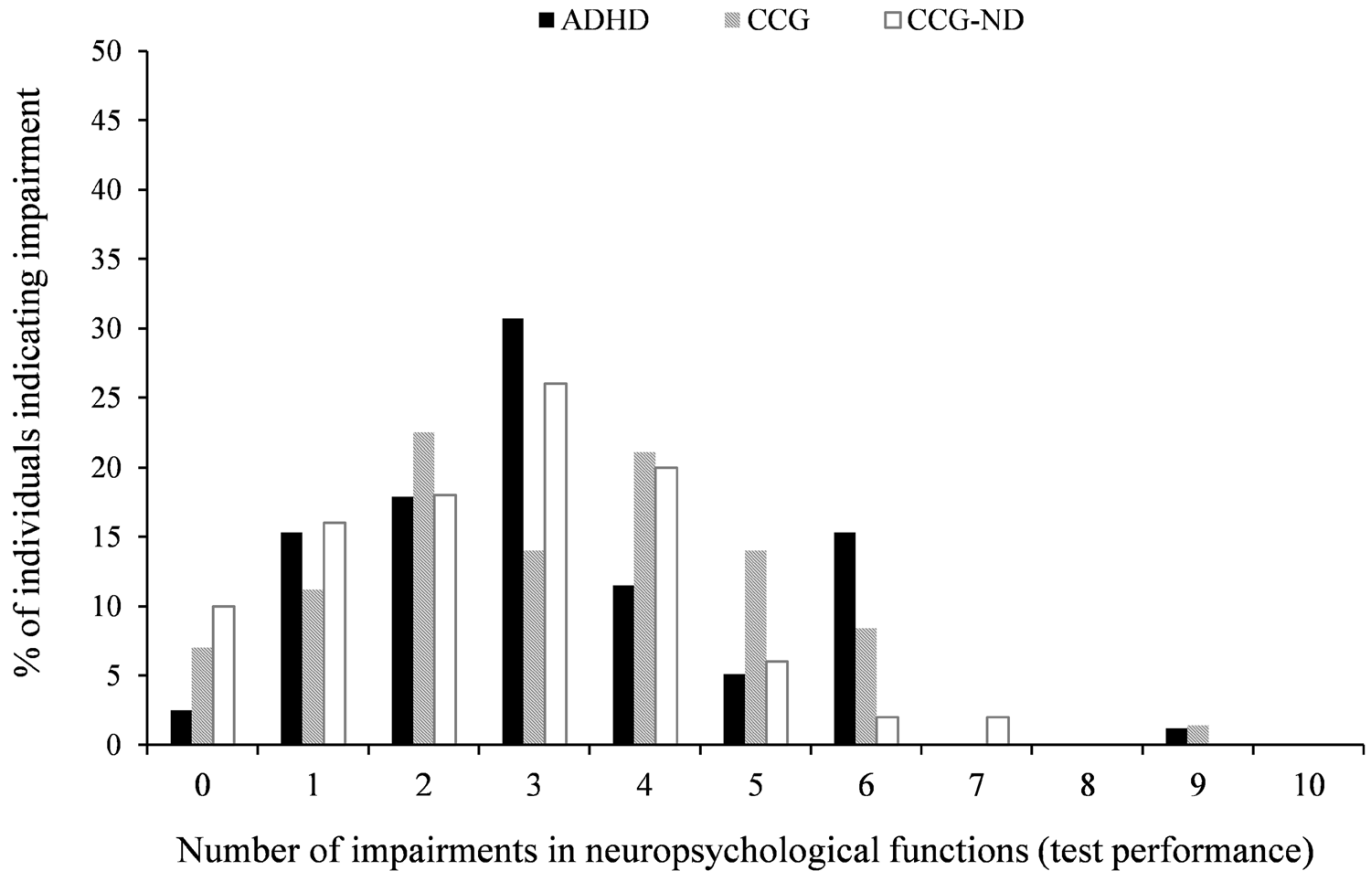
Percentage of individuals showing impairment in neuropsychological functions, ranging from 0 (no impairment) to 10 (impairment in ten functions). *ADHD* attention deficit hyperactivity disorder; *CCG* clinical comparison group; *CCG-ND* clinical comparison group-not diagnosed

Descriptive statistics of neuropsychological test performance as well as the percentage of impairment per test variable and neuropsychological function are presented in Table 2 and Fig. 1. Decreased cognitive functions were found in all three groups compared to test norms, with a considerable proportion of individuals being impaired in aspects of attention, i.e., selective attention (52.0, 54.9, and 62.8% for the CCG-ND, CCG, and ADHD, respectively) and vigilance (49.0, 51.4, and 60.8%, respectively), inhibition (40.8, 39.1, and 49.3%, respectively), and interference control (30.6, 40.6, and 41.1%, respectively). Furthermore, the majority of individuals reported that they experience cognitive complaints in their daily lives (71.4, 95.5, and 89.2%, for the CCG-ND, CCG, and ADHD, respectively). The number of neuropsychological functions indicating impaired performance (Fig. 2) differs largely across individuals, with the majority of individuals (98.0, 98.6, and 98.8%, for the CCG-ND, CCG, and ADHD, respectively) having either no impairment or impairments in up to six functions.

**Table 2:** Neuropsychological test performance and self-report of the groups ADHD, CCG, and CCG-ND

Neuropsychological variables	ADHD					CCG					CCG-ND				
	Range (min–max)	Mean	Median	SD	% Percentile ≤ 16^a^	Range (min–max)	Mean	Median	SD	% Percentile ≤ 16^a^	Range (min–max)	Mean	Median	SD	% Percentile ≤ 16^a^
Selective attention^b^—Logarithmic mean of RT	159–621	359	350	79	15.5	149–739	375	368	94	18.3	49–563	358	348	86	14
Selective attention^b^—Logarithmic SD of RT	0–5.43	1.43	1.28	0.80	54.5	1.09–9.08	1.41	1.25	0.97	36.6	1.11–9.82	1.43	1.25	1.21	40
Selective attention^b^—Omission errors	0–30	1.46	0	4.16	33.7	0–12	0.54	0	1.69	22.5	0– 2	0.34	0	0.59	28
Vigilance^c^—Logarithmic mean of RT	253–688	458	458	76	16.4	307–706	462	447	82	19.7	341–726	457	459	71	8.2
Vigilance^c^—Omission errors	0–21	3.41	2	4.04	59.5	0–13	2.50	1	3.21	46.4	0– 18	2.73	1	3.79	48.9
Speed of processing^d^—Time needed in seconds	2.2–37	21.11	20.30	5.73	22.1	13.5–43.1	23.47	23.1	5.99	32.3	11.1–34.7	20.43	19.9	4.71	10
Working memory^e^—Correct responses	0–15	10.91	12	3.49	22.1	0–23	11.23	12	4.01	16.9	0–15	11.02	12	3.73	24
Figural fluency^f^—Unique patterns created	11–51	25.38	24	9.13	22.1	6–46	22.39	20	8.74	29.5	10–49	25.56	23	9.56	18
Interference^g^—Reading interference	− 0.03–1.1	0.22	0.18	0.17	38.3	− 0.04–0.86	0.20	0.17	0.15	30.4	− 0.04–0.49	0.17	0.14	0.13	24.5
Interference^g^—Naming interference	− 0.01–2.78	0.15	0.11	0.33	10.9	− 0.07–0.46	0.14	0.11	0.10	17.1	0.02–0.46	0.14	0.13	0.08	12.2
Planning ability^h^—Number of items solved	7–22	13.49	14	3.04	17.2	7–22	14.45	15	3.44	14.9	8–21	15.02	15	3.64	14.3
Inhibition^i^—Commission errors	2–32	15.79	15	7.85	49.3	2–32	13.45	12	7.32	39.1	2–30	13.51	12	7.14	40.8
Flexibility^j^—Quotient score	0.76–3.85	1.58	1.50	0.47	10.5	0.95–4.67	1.61	1.52	0.54	18.3	0.62–2.62	1.48	1.41	0.35	8.3
Task switch accuracy^k^—Accuracy score	− 8–20	2.80	2	5.84	25.3	− 11–18	3.70	3	5.29	22.7	− 10–32	3.90	3	6.50	22
Subjective experiences of cognitive functioning ^l,1^—Impairment score	19–120	77.24	83	21.44	89.2	4–106	77.18	78	17.37	95.5	23–93	62.97	66.50	20.68	71.4

*ADHD* attention deficit hyperactivity disorder; *CCG* clinical comparison group; *CCG-ND* clinical comparison group-not diagnosed

^a^Percentage of individuals with percentile rank ≤ 16 based on test norms. ^b^Perceptual and Attention Functions-Selective Attention (WAFS). ^c^Perceptual and Attention Functions—Vigilance (WAFV). ^d^Trail-Making Test-A (TMT-A). ^e^N-back verbal task (NBV). ^f^5-Point Test—Langensteinbach Version. ^g^Stroop Interference Test. ^h^Tower of London—Freiburg Version (TOL-F). ^i^Go/No-Go test. ^j^Time to complete TMT-B divided by time to complete TMT-A. ^k^SWITCH task. ^l^Questionnaire on Mental Ability (FLEI). ^1^ADHD group reported significantly more problems of cognitive functioning than CCG-ND

Nonparametric group comparisons (Mann–Whitney *U* Tests) were computed to determine performance differences between the ADHD group and both the CCG and the CCG-ND. Test statistics of Table 3, as well as bar charts presenting the number of impairments in Figs. 1 and 2, indicate no meaningful group differences. The only significant effect of medium size was observed in subjective experiences of cognitive functioning, i.e., the ADHD group reported significantly more problems of cognitive functioning in their daily lives than the CCG-ND. In addition, some effects did not reach significance but indicate a trend level effect of small size (Table 3). Specifically, when compared to the CCG, the ADHD group performed faster in the TMT-A and better in the figural fluency task (5-point test), but showed worse planning ability (TOL-F). When compared to the CCG-ND, the ADHD group showed better naming interference ability in the Stroop task, but worse planning ability in the TOL-F. However, differences in processing speed between groups must be interpreted with caution, because groups differed substantially in age, and age was observed to be significantly associated to processing speed in medium to large-sized correlations, i.e., *r* = 0.31, *r* = 0.34, and *r* = 0.26, for CCG-ND, CCG, and ADHD, respectively.

**Table 3:** Comparison of neuropsychological functions between ADHD, CCG, and CCG-ND

Neuropsychological variables	Group comparison^a^					
	ADHD vs. CCG			ADHD vs. CCG-ND		
	*Z*	*P*	*Cohen’s r*^b^	*Z*	*P*	*Cohen’s r*^b^
Selective attention^c^—Logarithmic mean of RT	− 0.87	0.38	− 0.07	− 1.38	0.89	+ 0.12
Selective attention^c^—Logarithmic SD of RT	− 1.67	0.09	+ 0.14	− 1.48	0.14	+ 0.13
Selective attention^c^—Omission errors	− 1.67	0.09	+ 0.14	− 1.05	0.29	+ 0.09
Vigilance^d^—Logarithmic mean of RT	− 0.21	0.83	+ 0.02	− 0.15	0.88	+ 0.01
Vigilance^d^—Omission errors	− 1.70	0.09	+ 0.14	− 1.39	0.16	+ 0.13
Speed of processing^e^—Time needed in seconds	− 2.45	0.014	− 0.20	− 0.71	0.48	+ 0.06
Working memory^f^—Correct responses	− 0.72	0.47	+ 0.06	− 0.41	0.68	− 0.04
Figural fluency^g^—Unique patterns created	− 1.92	0.05	− 0.16	− 0.11	0.91	+ 0.009
Interference^h^—Reading interference	− 0.30	0.77	+ 0.03	− 1.59	0.11	+ 0.15
Interference^h^—Naming interference	− 1.26	0.21	− 0.11	− 2.02	0.04	− 0.18
Planning ability^i^—Number of items solved	− 1.95	0.05	+ 0.17	− 2.32	0.02	+ 0.20
Inhibition^j^—Commission errors	− 1.77	0.07	+ 0.15	− 1.54	0.12	+ 0.14
Flexibility^k^—Quotient score	− 0.05	0.96	− 0.004	− 0.88	0.38	− 0.08
Task switch accuracy^l^—Accuracy score	− 1.12	0.26	− 0.09	− 0.98	0.33	+ 0.09
Subjective experiences of cognitive functioning^m^—Impairment score	− 0.31	0.76	− 0.03	− 3.44	0.001**	+ 0.33

*ADHD* attention deficit hyperactivity disorder; *CCG* clinical comparison group; *CCG-ND* clinical comparison group-not diagnosed

**Statistically significant at *p* < .01

^a^Mann–Whitney *U* test. ^b^Positive values indicate worse functioning in ADHD in the respective comparison, negative values indicate better functioning in ADHD in the respective comparison. ^c^Perceptual and Attention Functions-Selective Attention (WAFS). ^d^Perceptual and Attention Functions- Vigilance (WAFV). ^e^Trail-Making Test-A (TMT-A). ^f^N-back verbal task (NBV). ^g^5-Point Test—Langensteinbach Version. ^h^Stroop Interference Test. ^i^Tower of London—Freiburg Version (TOL-F). ^j^Go/No-Go test. ^k^Time to complete Trail Making Test-B (TMT-B) divided by time to complete Trail Making Test-A (TMT-A). ^l^SWITCH task. ^m^Questionnaire on Mental Ability (FLEI)

Spearman’s rank correlation coefficients between basic and complex aspects of cognitive functions in the three groups are presented in Table 4. For the ADHD group, a small-sized effect was found between basic cognitive functions and the total compound score of complex cognitive functions (*r* = 0.28). Differentiating between different aspects of complex cognitive functions, a significant association of medium size was obtained with inhibition/interference control (*r* = 0.36) and a significant association of small size with fluency (r = 0.29). For the CCG, a significant and medium-sized effect was found for the total compound score of complex cognitive functions (*r* = 0.34), with a significant small-sized effect to inhibition/interference control (*r* = 0.26) and a significant medium-sized effect to fluency (*r* = 0.32). Finally, a small and nonsignificant association was found between basic cognitive functions and the compound score of complex cognitive functions of the CCG-ND (*r* = 0.15). Per domain of complex cognitive functions, a significant effect (medium size) was revealed only for the association with fluency (*r* = 0.45).

**Table 4:** Correlation coefficients (Spearman rank correlation) between basic cognitive functions and different aspects of complex cognitive functions in ADHD, CCG, and CCG-ND

Complex cognitive functions	Spearman’s *r* (*p*)		
	ADHD	CCG	CCG-ND
Working memory	0.138 (*p* = 0.232)	0.161 (*p* = 0.180)	− 0.041 (*p* = 0.780)
Inhibition/interference control	0.355** (*p* = 0.002)	0.264* (*p* = 0.027)	− 0.020 (*p* = 0.893)
Cognitive flexibility	0.018 (*p* = 0.873)	0.005 (*p* = 0.964)	− 0.206 (*p* = 0.152)
Fluency	0.292* (*p* = 0.010)	0.322** (*p* = 0.006)	0.449** (*p* = 0.001)
Planning	0.156 (*p* = 0.222)	0.209 (*p* = 0.089)	0.143 (*p* = 0.325)
Total compound	0.282* (*p* = 0.012)	0.344** (*p* = 0.003)	0.146 (*p* = 0.311)

Basic cognitive functions: Compound *Z*-score of selective attention task (logarithmic mean of RT, logarithmic standard deviation of RT, omissions), vigilance task (logarithmic mean of RT and omissions), and TMT part A; Working memory: *Z*-score of correct responses in NBV; Inhibition/Interference control: Compound *Z*-score of Go/No-Go omissions and Stroop Interference Test naming interference and reading interference; Cognitive flexibility: Compound *Z*-score of TMT part B/TMT part A and SWITCH task switch accuracy; Fluency: *Z*-score of number of unique patterns created in 5-Point Test; Planning: *Z*-score of number of items solved in TOL-F; Total compound: Compound *Z*-score of working memory, inhibition/interference control, cognitive flexibility, fluency, and planning

*ADHD* attention deficit hyperactivity Disorder; *CCG* clinical comparison group; *CCG-ND* clinical comparison group-not diagnosed

*Significant at the 0.05 level. **Significant at the 0.01 level

## Discussion

This study aimed to explore neuropsychological functioning of individuals at clinical evaluation of adult ADHD, and examine the associations between basic and higher-order cognitive functions in this population. An analysis of neuropsychological test performance revealed that individuals with ADHD exhibit impairments in several of the neuropsychological functions assessed in this study. Considerable rates of impairment, as determined by use of test norms, were shown in adults with ADHD in selective attention, vigilance, inhibition, and interference control (63, 61, 49, and 41% of participants, respectively). This is in line with the results of numerous previous studies showing impairments in adults with ADHD in various cognitive functions (Bálint, Czobor, Mészáros, Simon, and Bitter 2008; Boonstra et al. 2005; Pritchard, Neumann, and Rucklidge 2008). The present results conform to previous findings, demonstrating that slower responses, a greater reaction time variability, and more omission were commonly observed in adults with ADHD when compared to healthy control participants in tests of attention (Cross-Villasana et al. 2015; Kofler et al. 2013; Mostert et al. 2015). The sensitivity of the vigilance task to reveal cognitive impairment underlines its central role in the neuropsychological assessment of adult ADHD, despite its long administration time may cost comparably much clinical resources. Furthermore, this study demonstrates marked cognitive complaints as reported by patients with ADHD. The pronounced experiences of cognitive impairments in daily life activities have been reported in earlier research on adults with ADHD (In de Braek et al. 2011; Fuermaier et al. 2014), and may also explain the referral reason of the present sample, as all individuals were seeking a clinical evaluation of adult ADHD as they thought to experience ADHD-like problems in their daily lives. When comparing neuropsychological studies in ADHD across lifespan, it becomes apparent that ADHD is characterized by heterogeneous cognitive profiles with marked differences between individuals, but also across time (Luo, Weibman, Halperin and Li 2019; Seidman 2006). For example, neuroimaging studies demonstrated morphological and physiological changes in ADHD over time to be associated with differences in neuropsychological functioning (Cortese et al. 2012; Hoogman et al. 2017; Krain, and Castellanos 2006). Furthermore, potential comorbid disorders that individuals with ADHD may grow into in adolescence and early adulthood, as well as drug abuse that often commences in this development phase, are likely to represent additional sources for marked inter-individual differences in neuropsychological profiles in young adults with ADHD (Marks, Newcorn and Halperin 2001; Rose, Bramham, Young, Paliokostas and Xenitidis 2009).

Moreover, the present study demonstrates that individuals of the clinical comparison groups, i.e., the CCG and CCG-ND, showed a similar pattern of neuropsychological functioning and exhibit impairments in the same functions as observed in the group of patients diagnosed with ADHD, including selective attention, vigilance, inhibition, interference control, as well as in subjective ratings of cognitive functioning. This is also illustrated by an inspection of the number of impairments by group, which shows a similar distribution for the ADHD group, CCG, and CCG-ND. The vast majority of individuals have impairments in one to six functions (of ten functions assessed), with a peak at two to four impairments. The observation of similar patterns of neuropsychological functions between the three groups is consistent with the view of ADHD as dimensional construct, with ADHD-like symptoms and impairments occurring in large parts of the population, including the general psychiatric population (Sergeant, Geurts and Oosterlaan 2002). In this context, multifactorial models are discussed in the etiology of ADHD, with, for example, a large number of gene loci that may contribute to the clinical syndrome of ADHD (Bobb et al. 2005; Cortese 2012; Li et al. 2006; Demontis et al. 2019). The notion of a similar pattern of neuropsychological functioning across the three groups is supported by group comparisons revealing mostly non-significant group differences, ranging from negligible to small size. A significant difference between groups was found in the subjective experience of cognitive functioning only. In this self-report, patients with ADHD indicate significantly more pronounced cognitive complaints compared to the CCG-ND. However, inspecting the magnitude of cognitive complaints of all three groups, it becomes apparent that the complaints may be no good indicator for differential diagnostic purpose, as pronounced and marked cognitive impairments are reported by all three groups at clinical assessment, which may explain their referral to an ADHD outpatient clinic. Taken together, data of this study, on the one hand, provide evidence for the notion that a neuropsychological assessment may have limited ability to discriminate between adult ADHD and other psychiatric disorders in a psychiatric assessment (Barkley 2019; Holst and Thorell 2017; Pettersson et al. 2018; Solanto, Etefia and Marks 2004; Walker et al. 2000). On the other hand, marked cognitive impairments that are observed in the majority of individuals with ADHD in this study supports earlier seminal work which argued that a neuropsychological assessment using cognitive performance tests may contribute to the comprehensive understanding of an individual, including the characterization of individual cognitive strengths and weaknesses and potentially also guide treatment planning, such as the administration of cognitive remediation programs or acquiring compensation strategies to overcome consequences of cognitive deficits (Lange et al. 2014; Mapou 2019; Pineda et al. 2007). Regarding cognitive remediation, there is yet an ongoing discussion on its usefulness in the treatment of adults with ADHD with nonconforming findings reported in different studies (Chevalier et al. 2017; Cortese et al. 2015; Rapport, Orban, Kofler and Friedman 2013; Solanto, Marks, Mitchell, Wasserstein and Kofman 2008). Further research is therefore needed on the extent to which neuropsychological tests can effectively be used to guide psychological interventions.

Finally, findings of earlier research (Butzbach et al. 2019; Holst and Thorell 2017) could be confirmed by demonstrating significant correlations between basic cognitive functions and higher-order cognitive functions in the ADHD group. The observed associations support the impairments in basic functions may lead to impairments in higher-order functions, such as aspects of complex attention and executive control. Noteworthy, the present study adds to previous research in demonstrating significant and medium-sized associations between basic and higher-order cognitive functions not only in the ADHD group but also in the CCG. This effect may indicate that the relationship between basic and higher-order cognitive functions may not be specific for adult ADHD, but may also hold true in individuals with other psychiatric disorders. In contrast, no such relationship was found in the clinical comparison group with no diagnostic status. The findings of a hierarchical relationship between basic and higher-order cognitive functions may not only be utilized to optimize neuropsychological assessment, but provides also implications for the treatment of cognitive deficits of patients with psychiatric conditions. Previous research already demonstrated that stimulant drug treatment improves basic cognitive functions, i.e., processing speed and reaction time variability, which in turn may indirectly improve higher-order cognitive functions (Bron et al. 2014; Butzbach et al. 2019; Kofler et al. 2013; Wong and Stevens 2012). Similarly, cognitive remediation programs aiming to improve processing speed and other aspects of basic attention may have a broader area of effect than initially assumed, and may also impact on higher-order functions (Sonuga-Barke et al. 2014).

## Limitations

This study needs to be seen in the context of several limitations. First, the group of patients with ADHD is a selected sample, with the majority being diagnosed with the combined symptom presentation and various comorbid psychiatric disorders. It is, therefore, difficult to evaluate how representative the present data are for the population of adults with ADHD when compared to clinical control groups, and whether the observed effects would hold in clinical samples with different characteristics.

Second, because the clinical assessment was designed mainly to determine the presence of adult ADHD, only clinical indications, but no verified diagnoses, could be given for the differentiation between other clinical conditions. Thus, the differentiation between individuals presumably having or not having psychiatric conditions, and subsequent group comparisons, must be interpreted with caution.

Third, the missing of more differences between groups may have been caused by similarities in group characteristics. For example, a similar range of psychiatric disorders is observed both in the ADHD group and the CCG. Further, given this context of an ADHD outpatient clinic, it must be considered that also individuals not being diagnosed with ADHD may suffer from a similar clinical pattern which may just not reach diagnostic threshold for ADHD.

Fourth, even though the neuropsychological assessment using cognitive tests was not part of the standard diagnostic routine of clinicians, results of the cognitive assessment were accessible to patients and clinicians, and may have guided clinical decision-making. However, this may even support the notion that a neuropsychological assessment using cognitive tests may not contribute substantially to a differential diagnostic process of psychiatric disorders, if one takes into account that the neuropsychological assessment was not completely independent from the diagnostic assessment and still the ADHD group does not largely differ in neuropsychological functions from the two other clinical groups.

## Conclusions

This study demonstrates that individuals seeking a clinical evaluation of adult ADHD show marked impairments in several aspects of cognitive functions, irrespectively from whether they fulfill diagnostic criteria for ADHD or not. This is underlined by group comparisons indicating no meaningful differences in cognitive functions between patients with ADHD, the clinical comparison group, and the clinical comparison group with no diagnostic status. We conclude that cognitive deficits are prominent in patients of this setting, but are not specific for ADHD. And a neuropsychological assessment using cognitive tests may not provide the clinician with incremental information for the differential diagnostic process of adult ADHD. Furthermore, we conclude and support earlier work that deficits in a range of cognitive domains can be substantially explained by deficits in lower-order cognitive functions, such as processing speed and basic aspects of attention and distractibility.
